# Bioinformatics: A rational combine approach used for the identification and *in-vitro* activity evaluation of potent *β-Glucuronidase* inhibitors

**DOI:** 10.1371/journal.pone.0200502

**Published:** 2018-12-05

**Authors:** Maria Yousuf, Nimra Naveed Shaikh, Zaheer Ul-Haq, M. Iqbal Choudhary

**Affiliations:** 1 Dr. Panjwani Center for Molecular Medicine and Drug Research, International Center for Chemical and Biological Sciences, University of Karachi, Karachi, Pakistan; 2 H. E. J. Research Institute of Chemistry, International Center for Chemical and Biological Sciences, University of Karachi, Karachi, Pakistan; 3 Department of Biochemistry, Faculty of Science, King Abdulaziz University, Jeddah, Saudi Arabia; Chuo University, JAPAN

## Abstract

Identification of hotspot drug-receptor interactions through *in-silico* prediction methods (Pharmacophore mapping, virtual screening, 3DQSAR, etc), is considered as a key approach in drug designing and development process. In the current design study, advanced *in-silico* based computational techniques were used for the identification of *lead-like* molecules against the targeted receptor *β*-*glucuronidase*. The binding pattern of a potent inhibitor in the ligand-receptor X-ray co-crystallize complex was used to identify and extract the structure-base Pharmacophore features. Based on these observations; five structure-based pharmacophore models were derived to conduct the virtual screening of ICCBS *in-house data-base*. Top-ranked identified Hits (33 compounds) were selected to subject for *in-vitro* biological activity evaluation against *β-glucuronidase* enzyme; out of them, twenty compounds (61% of screened compounds) evaluated as actives, however eleven compounds were found to have significantly higher inhibitory activity, including compounds **1**, **5**–**8**, **10**, **12**–**13**, and **17**–**19** with IC_50_ values ranging from 1.2 μM to 34.9 μM. Out of the eleven potent inhibitors, seven compounds **1**, **5**, **6**, **7**, **8**, **13**, and **19** were found new, and evaluated first time for the *β-glucuronidase* inhibitory activity. Compounds **1**, **5** and **19** exhibited a highly potent inhibition in uM of *β-glucuronidase* enzyme with non-cytotoxic behavior against the mouse fibroblast (3T3) cell line. Our combined *in-silico* and *in-vitro* results revealed that the binding pattern analysis of the eleven potent inhibitors, showed almost similar non-covalent interactions, as observed in case of our validated pharmacophore model. The obtained results thus demonstrated that the virtual screening minimizes false positives, and provide a template for the identification and development of new and more potent *β-glucuronidase* inhibitors with non-toxic effects.

## Introduction

*β-Glucuronidase* belongs to the glycosidase family of enzymes, which catalyze the hydrolysis of complex carbohydrates. The active site of the enzyme consists of a large cleft at the interface of two monomeric units. Two acidic amino acids, *i*.*e*., Glu 540 and Glu 451, and one aromatic amino acid, *i*.*e*., Tyr 504, have been proposed to be important for catalysis [1–2]. Human *β-glucuronidase* is homologous to the *Escherichia coli* enzyme *β-glucuronidase*. It catalyzes the hydrolysis of carbohydrates using two acidic a.a residues, Glu 540 and Glu 451. Additionally, the a.a residue Tyr 504 is involved in this catalytic event [3]. The catalytic mechanism involves three steps, as follows (1) Nucleophilic attack of the carboxylate anion on the anomeric carbon of sugar, (2) Hydrolysis of glucuronyl enzyme intermediate, and (3) De-glucuronidation [4–5].

Over-expression of *β-glucuronidase* enzyme activity is associated with several disorders, including various types of cancers, particularly hormone-dependent cancers, such as breast, prostate, and colon cancers. For the treatment of disorders associated with increased *β-glucuronidase* activity, d-saccharic acid 1, 4-lactone (DSL; saccharo lactone), silymarin, and silybin (crude drugs) are commercially available [6–7]. However, these drugs decreases immunity, and cause adverse effects. Therefore, there is a strong need to develop new *β-glucuronidase* inhibitors with improved potency and fewer adverse effects.

Structure-based pharmacophore mapping considered as a useful tool for medicinal chemists to identify novel ligands that have a high probability of being biologically active. This method utilizes the following steps: (I) Protein structure preparation, (II) Binding site detection, (III) Pharmacophore features identification, and (IV) Pharmacophore features selection.

Structure-based Pharmacophore can be efficiently used for virtual screening, ligand-receptor binding pose prediction, and binding site similarity search. Therefore, this method is a valuable tool for Hit and lead optimization, compounds library design, scaffold hopping, virtual screening, and multi-target drug design [8–10]. A successful virtual screening can identify molecules with novel chemical structural features that bind to the target receptor of interest in a large chemical space (*e*.*g*. the needle in a haystack concept).

**Fig 1:**
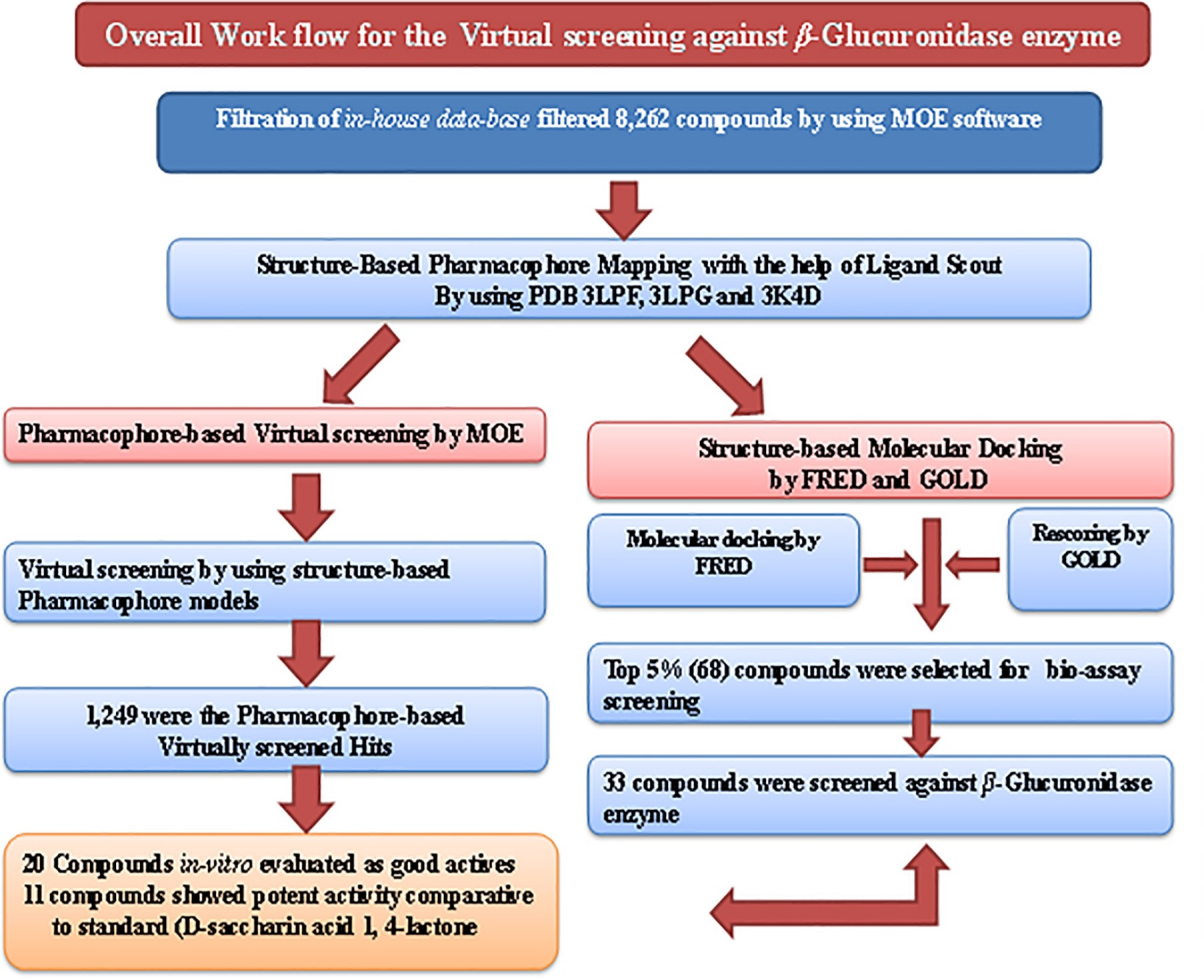
Overall schematic work flow representation. The structure-based Pharmacophore mapping, Virtual screening and *in-vitro* biological activity evaluation of ICCBS *in-house data-base* against *β-Glucuronidase* enzyme.

The main purpose of our entire designed study was to develop structure-based Pharmacophore models, extracted with appropriate chemical structural features information, and use of those models to conduct the virtual screening of an *in-house data-base* in search of new lead candidates as inhibitors of *β-glucuronidase* with more potency [Fig 1]. For this purpose, we used advance *in-silico* techniques of computer-aided drug design (CADD) to reduce the large chemical space, and to increase the focus on more promising candidates towards lead discovery and optimization.

## Results

### Pharmacophore-based virtual screening

Pharmacophore-based virtual screening provides a comprehensive and sophisticated method to screen millions of compounds *data-base* within a manageable time frame. In this way, virtual screening is expected to play a vital role in future rational drug design processes. In the present study, software derived models [11] were used to search the chemical *data-base* of ICCBS, which consisted of **8,262** filtered structurally diverse molecules, by using the software Molecular Operating Environment MOE (2010–212)[12], [S1 Appendix ].

**Fig 2:**
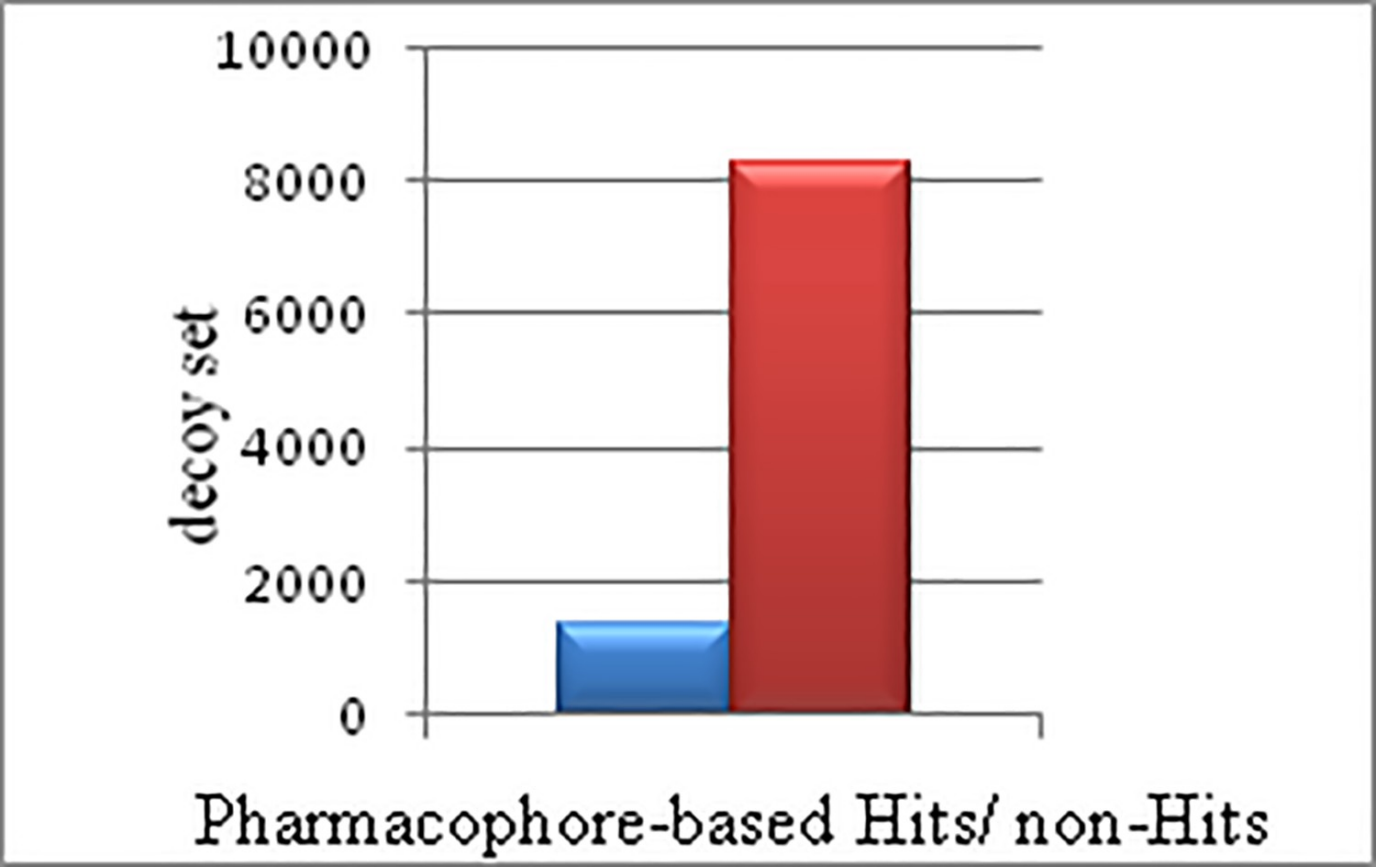
Graphical representation of Pharmacophore-based Hits / non-Hits with decoys. The **X-**axis (blue bar-graph) represents total number of Pharmacophore-based Hits (**1,249**) compounds redundancy of *in-house data-base*, while the red bar-graph depicting total number of compounds non-redundancy in decoys. The **Y**-axis represents total number of screened compounds in the decoy-set (**8,262**) of *in-house data-base*.

The software used Pharmacophore models and searched the query editor in the provided *data-base*. Pharmacophore-based virtual screening identified **999** Hits through shared and merged feature Pharmacophore models, **65** Hits were obtained by using 3LPF individual model, **85** Hits were obtained by using 3LPG individual Pharmacophore model, and finally **100** Hits were obtained through the individual 3K4D Pharmacophore model; thus a total of **1,249** Pharmacophore-based Hits were identified [**Fig 2**].

### Molecular docking results of Pharmacophore-based Hits

**Fig 3:**
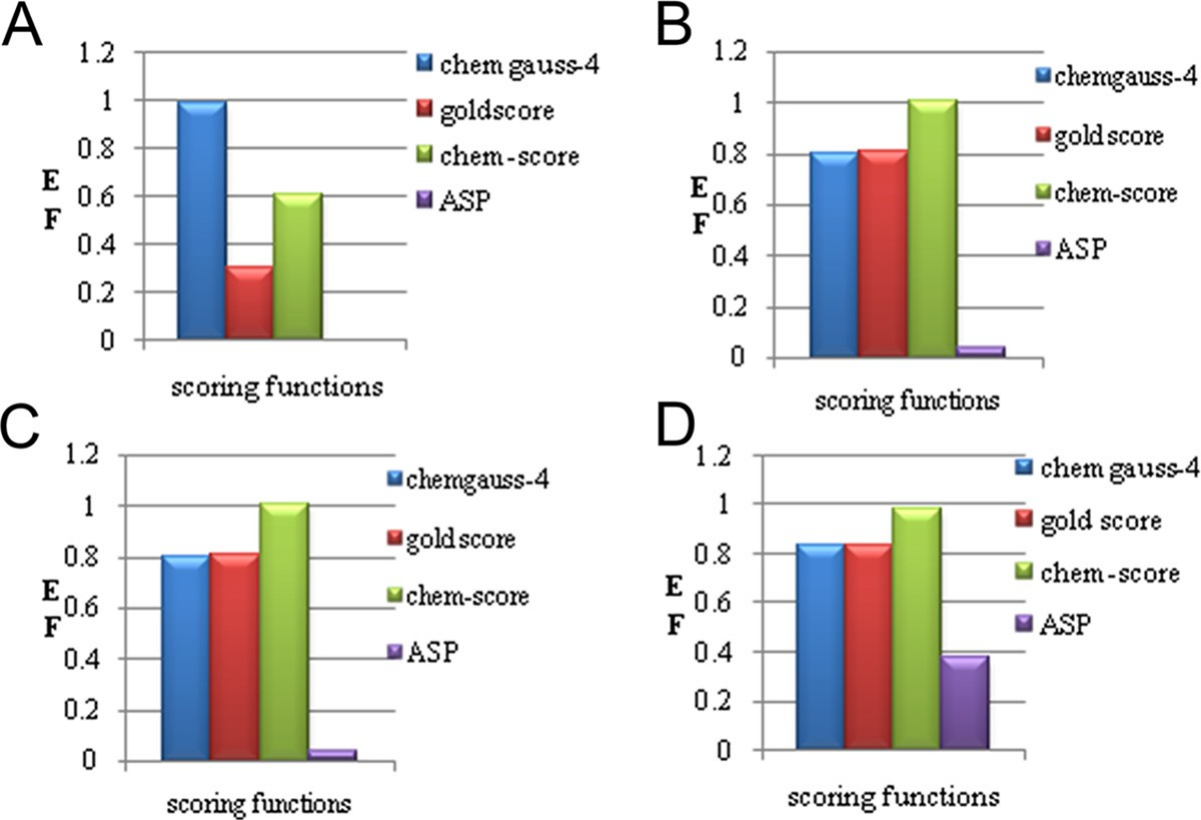
(**A**)The bar-graph illustrated the enrichment factors, **for 5%** of *data-base* in which chemgauss-4 scoring function of *FRED* software is dominant represented with (blue bar), (**B-D**) For the remaining **10%, 15%** and **20%** of *data-base* scoring function chem score of *GOLD* software is dominant showed with (orange bar).

Molecular docking studies of pharmacophore-based Hits was carried out along with **66** previously reported inhibitors from literature (belonging to different classes of compounds) [13] [S1 *Data-set*] by using Fast Rigid Exhaustive Docking (FRED) software [14–17]. Rescoring of the chemgauss-4 scoring function of FRED software was performed by using GOLD docking software. Enrichment factors were calculated for 5%, 10%, 15%, and 20% of screened *data-base* for each scoring function chemgauss-4, chem score, gold score, and ASP score, respectively [Fig 3A–3D] to examine the potential strength of all scoring functions for identifying *drug-like* candidates (redundancy of the *in-house data-base*), and to ultimately remove the non-binders (non-redundancy of the decoy set) [18], [S1 Appendix].

#### Enrichment factors of FRED and GOLD scoring functions

The enrichment factors of screened *data-base* by software FRED and GOLD with scoring functions chemgauss-4, gold score, chem score, and ASP score [19–20], were calculated for 5% (4.96%), 10% (10.60), 15% (15.15%), and 20% (20.09%) respectively, [Table 1, Fig 3A–3D], [S1 Appendix].

**Table 1:** % Enrichment factor.

%Enrichment Factor	Chemgauss-4	Gold score	Chem score	ASP score
**5%**	**4.96%**	1.51%	3.03%	0%
**10%**	7.57%	6.05%	**10.60%**	6.69%
**15%**	12.10%	12.12%	**15.15%**	7.50%
**20%**	16.69%	16.69%	**20.09%**	12.21%

**Enrichment factor**: For **5%** of *data-base* scoring function chemgauss-4 of FRED software is dominant, while for rest of the **10%, 15%** and **20%** of *data-base* scoring function chem score of GOLD software is dominant among the all [Table 1].

#### Receiver operating characteristic (ROC) curves

ROC curves are used to validate the docking software performance, which differentiates between the true binders (true positives) and non-binders (false positives). ROC curves are the plots between sensitivity and (1-specificity), sensitivity defines the presence of true actives in a *data-base*. Higher the sensitivity values represent an increase number of true positives in the *data-base*, whereas specificity defines the presence of true-negatives (non-actives) in the *data-base*. The higher number of actives in the *data-set* represented the increased sensitivity and decreased probability of specificity (presence of non-binders). This statistical test is used in high-throughput computational-based virtual screening (HTS) for quick and efficient differentiation between the actives and non-actives in a decoy set of compounds. Based on this, we can consider an optimal model (true-binders) and reject (discard) sub-optimal model for data that is not required (comprised of non-binders).

Accuracy can be measured by using area under the curve (AUC). An area of **1** represents a perfect test, whereas an area of **0.5** is supposed to be worthless; however, an area of **0.7–0.9** is considered as an acceptable value.

**Fig 4:**
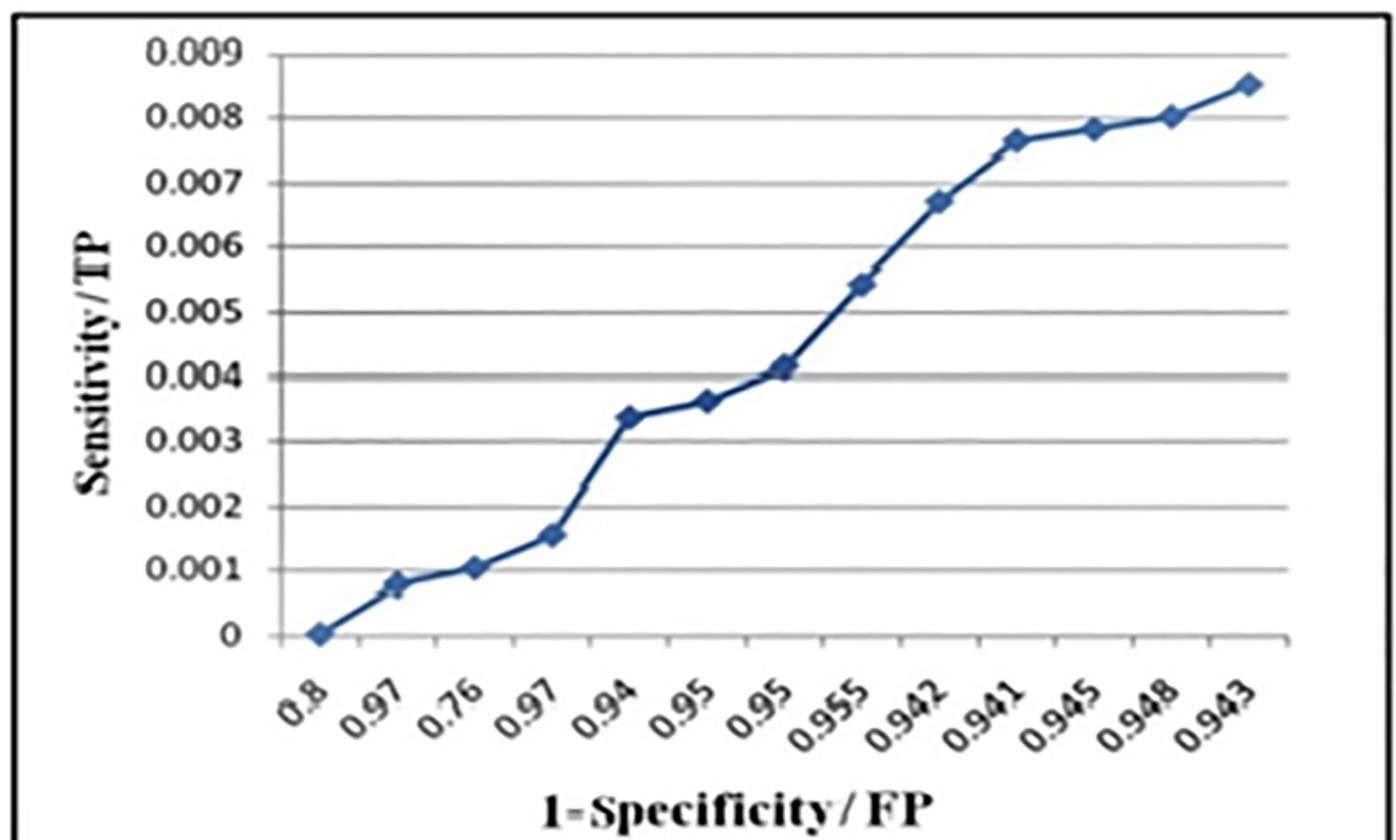
ROC curve between sensitivity and 1-specificity. Where, **X-axis** (1-specificity) represents the false positives in decoys. **Y-axis** (sensitivity) represents the (true +ves in the decoys).

In our case study, the AUC value calculated as **0.76**, which was a quite acceptable value, demonstrating that our *data-set* was considerably enriched with true binders (actives). Additionally, as we increased the cut off value of the *data-base* (small-subsets) in which true positives were present, the probability of finding TP increased, which ultimately raised the sensitivity [21–22], [Fig 4].

**Fig 5:**
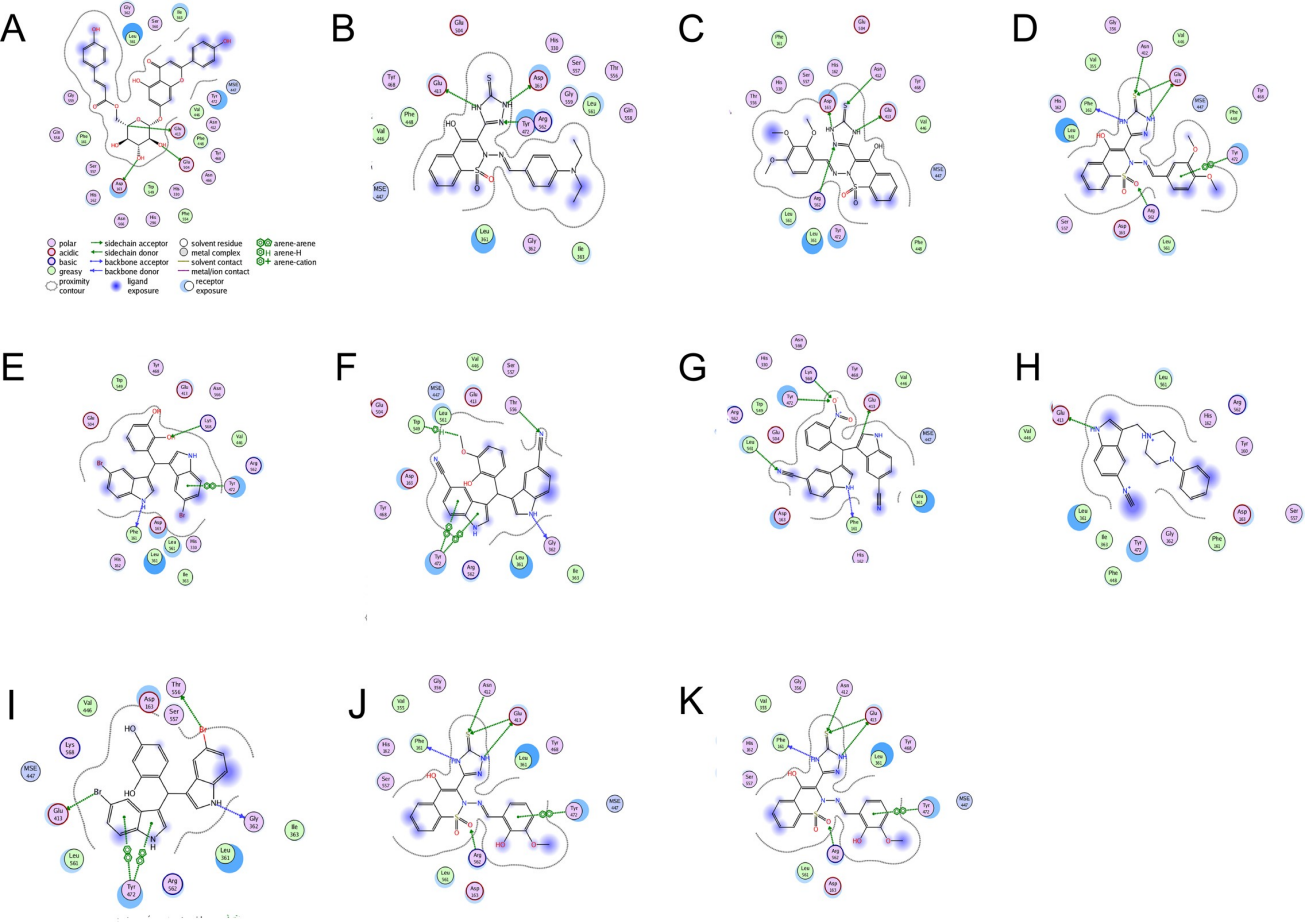
Binding pattern analysis of ligand-protein interactions with respect to IC_50_ values. For descriptions of figure parts A-K, see Table 2.

Out of the sixty eight compounds (top 5% enrichment of the virtually screened *data-set*), thirty three (33) compounds were selected for *in-vitro* bioassay screening to evaluate the hidden therapeutic potential against *β-glucuronidase* enzyme. Twenty compounds showed a good inhibitory potential. Whereas, eleven compounds were found to be more potent actives comparative to the standard (d-saccharic acid 1, 4-lactone; half-maximal inhibitory concentration [IC_50_] = 45.75 ± 2.16 μM). [Fig 5A–5K, Tables 2 and 3] (SAR).

**Table 2:** Binding pattern analysis of ligand-protein interactions with respect to IC_50_ values.

Compound #, Fig # and Molecular formula	Ligand-protein observed key non-covalent interactions Keys: HBD / HBA = Hydrogen bond donor and acceptor	Ligand-receptor docked poses derived by using MOE software	IC_50_ (μM) ±SEM
**(1)** **(5A)** **(C**_**30**_**H**_**26**_**O**_**12**_**)**	2-*β*-OH of pyranose substituent of the coumarin moiety acted as HB-donor to Glu 503, 3-*α*-OH acted as HB-donor to Asp 161 for H-bonding, while 5-*β*-H behaved as HB-donor to HB-acceptor Glu 413 for H-bonding.	**Interactive amino acid (a.a) residues** Glu 503 Asp 161 Glu 413	**4.50±0.44**
**(5)** **(5B)** **(C**_**21**_**H**_**22**_**N**_**6**_**O**_**3**_**S**_**2**_**)**	Thioimdazole (thione) moiety acted as HB-donor to HB-acceptor Asp163 making H-bonding while a.a Glu 413, Tyr 472 behaved as HB-donor to the HB-acceptor N atom for H-bonding.	**Interactive amino acid (a.a) residues** Asp 163 Glu 413 Tyr 472	**14.7±1.88**
**(6)** **(5C)** **(C**_**20**_**H**_**19**_**N**_**5**_**O**_**6**_**S**_**2**_**)**	One NH of triazole acted as HB-donor to a.a Glu 413 and formed H-bonding, while another NH behaved as HB-donor to Asp 163 for H-bonding, a.a Asn 412 acted as HB-donor to the sulfur atom and developed H-bonding while the a.a Arg 562 acted as HB-donor to the HB-acceptor lone pair of N atom for H-bonding.	**Interactive amino acid (a.a) residues** Glu 413 Asp 163 Asn 412 Arg 56	**11.8±0.86**
**(7)** **(5D)** **(C**_**19**_**H**_**17**_**N**_**5**_**O**_**5**_**S**_**2**_**)**	“S” of thioimidazole (thione) behaved as HB-acceptor from HB-donor a.a residue Asn 412 and Glu 413 respectively for H-bonding. NH of thioimidazole acted as HB-donor to HB-acceptor Phe 161 and Glu 413. However Arg 562 acted as HB-donor to the HB-acceptor sulphone group of compound. Amino acid Tyr 472 showed arene-arene, π-π stacking interactions with the di-methoxy substituted phenyl ring of the compound.	**Interactive amino acid (a.a) residues** Asn 412 Glu 413 Phe 161 Arg 562 Tyr 472	**11.41 ±0.04**
**(8)** **(5E)** **(C**_**23**_**H**_**16**_**Br**_**2**_**N**_**2**_**O**_**2**_**)**	NH- of indol ring behaved as backbone HB-donor to the HB-acceptor Phe 161 for H-bonding, another phenyl ring of bis-indol moiety showed arene-arene, π-π stacking interactions with a.a residue Tyr 472. While 2-Hydroxy substituted phenyl ring acted as HB-acceptor from a.a Lys 568 for H-bonding.	**Interactive amino acid (a.a) residues** Phe 161 Tyr 472 Lys 568	**1.20±1.03**
**(10)** **(5F)** **(C**_**26**_**H**_**18**_**N**_**4**_**O**_**2**_ **)**	Amino acid Tyr 572 showed arene-arene, π-π stacking interactions with indol phenyl ring. Trp 549 acted as arene-H donor to the HB-acceptor methoxy oxygen for H-bonding. Thr556 behaved as HB-donor to the HB-acceptor lone pair of azo-nitrogen.	**Interactive amino acid (a.a) residues** Tyr 472 Trp 549 Thr 556 Gly 362	**8.5±1.43**
**(12)** **(5G)** **(C**_**25**_**H**_**15**_**N**_**5**_**O**_**2**_**)**	Binding region amino acid residues Tyr 472, Lys 565 were acted as HB-donor to the oxygen atom of NO_2_ group substituted on phenyl ring to establish H-bonding, Leu 561 acted as HB-donor to the HB-acceptor lone pair of nitrogen atom of cyano group, while NH of indol ring behaved as HB-donor to the HB-acceptor Phe 161 for H-bonding.	**Interactive amino acid (a.a) residues** Tyr 472 Lys 568 Leu 561 Phe 161	34.9±0.21
**(13)** **(5H)** **(C**_**23**_**H**_**16**_**Br**_**2**_**N**_**2**_**O)**	NH of indol behaved as HB-donor to the HB-acceptor a.a Glu 413 for H-bonding within the binding region of receptor active site.	**Interactive amino acid (a.a) residue** Glu 413	**15.3±2.30**
**(17)** **(5I)** **(C**_**24**_**H**_**18**_**Br**_**2**_**N**_**2**_**O)**	Binding region promising aromatic amino acid residue Tyr 472 showed arene-arene, π-π stacking interactions with the phenyl and pyrol ring respectively, phenyl substituted with Br atom behaved as Lewis base (e-donor) to Glu 413 and formed H-bonding, while NH of indol moiety acted as HB-donor to HB-acceptor a.a Gly 362 to establish H-bonding.	**Interactive amino acid (a.a) residues** Tyr 472 Glu 413 Gly 362 Thr 556	**1.373±0.64**
**(18)** **(5J)** **(C**_**18**_**H**_**15**_**N**_**5**_**O**_**5**_**S**_**2**_**)**	NH- of indol ring behaved as backbone HB-donor to the HB-acceptor Phe 161 to develop H-bonding, while another phenyl ring of indol moiety showed arene-arene π-π stacking interaction with a.a residue Tyr472. 2-hydroxy substituent on phenyl ring acted as HB-acceptor from HB-donor Lys 568 a.a to establish H-bonding.	**Interactive amino acid (a.a) residues** **Tyr472** **Gly362** **Glu413** **Trp540** **Thr556**	**16.16±0.76**
**(19)** **(5K)** **(C**_**20**_**H**_**22**_**N**_**4**_^**2+**^**)**	One NH of triazole (thione) behaved as HB-donor to Glu 413, while another NH acted as HB-donor to HB-acceptor a.a Phe 161 for H-bonding, binding region a.a residues Asn 412 and Glu 413 acted as HB-donor to the HB-acceptor lone pair of sulfur atom and showed H-bonding. Amino acid Tyr 472 showed arene-arene, π-π stacking interactions with the phenyl ring.	**Interactive amino acid residues** Glu 413 Phe 161 Asn 412 Tyr 472 Arg 562	**16.65±0.69**

**Ligand-receptor non-covalent interactions pattern analysis:** within the provided 5Åof binding region, the most common interactions were observed with Glu 413, Tyr 472, and Phe 161 amino acid residues [Table 2 & Fig 5A–5K].

**Table 3:** Bio-assay screening results.

Compound no	% Inhibition	Conc. (μM)	IC_50_ μM±SEM	Compound no	% Inhibition	Conc. (μM)	IC_50_ μM±SEM
1	**96.9**	**200**	**4.5±0.44**	**12**	**57.3**	**50.0**	**34.9±0.21**
2	54.5	**200**	318.5±4.41	**13**	**73.4**	**400**	**15.3±2.30**
3	67.2	**200**	261.7±7.02	**14**	59.2	**200**	162.9±2.50
4	63.0	**200**	140.9±4.10	**15**	53.6	**200**	82.9±2.30
5	**95.1**	**200**	**14.7±1.88**	**16**	54.4	**200**	385.3±5.58
6	**81.0**	**200**	**11.8±0.86**	**17**	**99.2**	**200**	**1.30±0.64**
7	**78.1**	**200**	**11.4±0.04**	**18**	**75.9**	**200**	**16.1±0.76**
8	**97.8**	**200**	**1.2±1.03**	**19**	**93.4**	**200**	**16.6±0.69**
9	56.6	**200**	290.0±4.50	**20**	91.0	200	141.6±0.27
10	**62.1**	**200**	**8.5±1.43**				
11	67.4	**200**	94.4±1.30	Standard inhibitor*	**89.4**	**200**	**45.75±2.16**

***In-vitro* bio-assay screening results of twenty actives against *β-Glucuronidase* enzyme:** Compounds **1, 5–8, 10, 12–13** and **17–19** showed more potent inhibitory potential as compared to the standard (*D-saccharic acid 1, 4-lactone, IC_50_ = 45.75±2.16 μM).

#### Bio-assay screening results against *β-Glucuronidase* enzyme

For the biological activity evaluation of the top ranked 5% enriched compounds, identified by structure-based virtual screening of the *in-house data-base* using FRED software, 33 compounds were made available and subjected for *in-vitro* bio-assay screening against *β-glucuronidase* enzyme [S1 Appendix], [23]. Compounds **8**, (IC_50_ = 1.2 ± 1.03 μM) and **17**, (IC_50_ = 1.3 ± 0.64 μM) showed excellent potent inhibitory activity, whereas compounds **1**, **5**–**8**, **10**, **12–13**, **17**–**19**, with IC_50_ ranges in between 4.5 μM to 34.9 μM, were also showed remarkably potent inhibitory effect.

Compounds **11** and **15** showed a moderate inhibition, while compounds **2**–**4**, **9**,**11**, **14**–**16** and **20** showed weak inhibitory activity comparative to the standard (d-saccharic acid, 1,4-lactone; half-maximal inhibitory concentration (IC_50_ = 45.75 ± 2.16 μM) [Table 3], [S1 Appendix].

#### Cytotoxicity screening results

Eleven potent inhibitors of *β-glucuronidase* enzyme were also subjected for cytotoxicity assays [24] in 3T3 mouse fibroblasts cell line [S1 Appendix]. Out of the eleven screened compounds, three tested compounds were evaluated as completely non-cytotoxic [**1**, **5** and **19**], whereas the remaining eight compounds **6**–**8**, **10**, **12–13**, and **17–18**, showed moderate cytotoxicity comparative to the standard inhibitor (cycloheximide; IC_**50**_: 0.26 ± 0.1 μM), [Table 4].

**Table 4:** Cytotoxicity results.

Compound No.	Cytotoxicity (IC_50_ μ M± SEM)	
**1**	**>30**	
**5**	**>30**	
**6**	**17.13±1.41**	
**7**	**13.78±0.96**	
**8**	**16.84±0.99**	
**10**	**20.12±0.58**	
**12**	**9.56±0.13**	
**13**	**10.51±0.48**	
**17**	**9.53±0.32**	
**18**	**19.54±1.07**	
**19**	**>30**	
Standard*	**0.26±0.11**	

**Cytotoxicity results:** Compounds (**1, 5** and **19**) showed completely non-cytotoxic effect, while compounds (**6–8, 10, 12–13,** and **17–18**) exhibited moderately cytotoxic effect against the 3T3 mouse fibroblast cell line.

* (Cycloheximide).

### Structure-activity relationship (SAR)

#### SAR of halo (Cl, Br)-substituted indol derivatives

Halo-substituted bis-indols possess tremendous medicinal importance, [25] the docked pose interaction analysis of compound **8** demonstrated that H-bonding between the NH of indol and phenylalanine (Phe161) was responsible for its highly potent activity (**44** folds higher than the standard d-saccharic acid 1, 4-lactone), along with π-π stacking interactions between the active site amino acid Tyr 472 and the phenyl ring of indol. The presence of di-hydroxy substituted phenyl in compound **8**, one hydroxy group formed H-bonding with amino acid Lys 568. It was also responsible for the potent activity as compared to the activity of compound **17**. This could be explained by the absence of one OH group, which was replaced with one OMe (methoxy) group, exhibited H-arene interaction, a comparatively weaker interaction than the H-bonding. A decrease in the activity of compound **10** was observed due to the absence of one OH group, which was replaced with one OMe group, although this provided an electron donating group but it was not involved in H-bonding, as it was observed in compound **8**. Glu 413 a.a also acted as a hydrogen bond acceptor for compound **9** within the binding pocket region of 5 Å. The decrease in the activity of compound **18** was also observed due to the absence of H-bonding of one OH substituent in the phenyl ring [Tables 2 and 3].

#### SAR of cyclic thioimidazole derivatives

Docked pose interaction analysis of phenyl sulfone-substituted cyclic thiourea derivatives provided an insight into the most active compound **7**, from thioimidazole class which showed 78.1% inhibition,(IC_**50**_ = 11.41 ± 0.04 μM). In this compound, NH formed H-bonding with the active site amino acid residues Glu 413 and Phe 161, whereas the di-methoxy phenyl substituent showed π-π stacking interactions with Tyr 472. On the other hand, compound **6** with 81.0% inhibition, and (IC_**50**_ = 11.8 ± 0.86 μM), was slightly less potent than compound **7**. This decrease in activity was likely due to the absence of H-bonding between NH and Phe 161 and π-π stacking interactions with Tyr 472 in compound **6**. Compound **5** showed 95.1% inhibition, (IC_50_ = 14.7 ± 1.88 μM) in comparison to the above two compounds. A lower activity was observed due to the absence of the di-methoxy substituent on the phenyl ring therefore the π-π stacking interactions did not exist. A decrease in the activity of compound **19** was also observed due to the absence of one OMe substituent on the phenyl ring; thus, the only phenyl ring possess less electron donating effect comparatively substituted with OMe, as a consequence the strength of the existing π-π stacking interactions becomes weaker, resulting to decrease in the biological activity [Tables 2 and 3].

#### SAR of pyranose-substituted coumarin derivatives

The docked pose analysis of pyranose-substituted coumarin derivatives exhibited the binding pattern of the most active compound, among the three actives from coumarin class. Compound **1** showed 96.9% inhibition (IC_50_ = 4.5 ± 0.44 μM). The increase in activity showed due to H-bonding between the 5-*β*-H pyranose moieties with the active site amino acid residue Glu 413. This interaction was missing in compound **2** and **3**, which exhibited 67.2% inhibition, (IC_50_ = 261.7 ± 7.02 μM), and 54.5% inhibition, (IC_**50**_ = 318.5 ± 4.41 μM) respectively [Tables 2 and 3].

## Discussion

*β-Glucuronidase* is an important glycosidase enzyme with great biological importance, which provides space towards the libraries of small organic inhibitors designing [26–27]. It’s over expression is associated with several cancers [28]. Therefore to overcome the adverse effects associated with the available drugs, there is a strong need to search and identify lead candidates which possesses the therapeutic potential against this target receptor with less adverse effects. For this purpose we first derived structure-based pharmacophore models using Ligand Scout software 3.0 version. These developed models were furthermore used to search and identify the *drug-like* candidates from *in-house data-base* by using MOE software. The Pharmacophore based features exist at similar distances in the respective screened compounds of *data-base*, to picked up (query editor) searched for those compounds which have possibly the similar Pharmacophore match with our derived models, (Pharmacophore search on the bases of distances b/w the respective features of functional-groups). For conducting the virtual screening, ICCBS *in-house data-base* was used as decoy set, which comprises over 9,000 structurally diversified molecules d*ata-base* was initially filtered to obtain *drug-like* candidates by using MOE software filter, removed all of those compounds which deviate from “Lipinski rule of five” and follow drug ability criteria, initially unwanted, highly reactive, toxic and those compounds which deviate from (ROF), and possess poor bio-availability, were removed from the *data-base*, the filters ultimately selected those compounds which possess good ADME / Tox properties. Software Omega (Open Eye Scientific Software) was used for energy minimization through its MM2 Molecular Mechanics force field parameters for each compound of *data-base*, atom types were corrected and molecular charges were also added through SYBYL software [29–30]. Virtual screening of filtered compounds identified small organic molecules, which follows drug-ability criteria described by Rule of five (ROF). Therefore, computational based high-throughput screening (HTS) helped out to remove or eliminate toxic, larger, unstable and *non-drug-like* candidates from *data-base*, in the way restrict to identify *drug-like* candidates. As a result it prevents to identify higher molecular weight compounds (usually transition metal complexes, peptides etc) which sometimes appear active at the *in-vitro* level but suffer with bio-availability issues at the *in-vivo* level [31–32].

The major purpose of *in-silico* based analyses is to examine the existing non-covalent interactions with catalytic amino acid residues through molecular docking binding poses which showed a good efficiency by using the software and good enrichment in the *data-base* with sufficient active compounds [33]. These knowledge-based research findings were furthermore validated with the help of statistical methods, including ROC curves, AUC values, and enrichment factors, which have been successfully applied to identify true positives (true-binders), and efficiently remove the false positives (non-binders) in the *data-base*. Therefore, structure-based Pharmacophore model can be efficiently used to quick search and identify the Pharmacophore features in various diverse classes of compounds and considered as an alternative tool of molecular docking, however this cannot be observed in the ligand-base Pharmacophore model which lacks the interaction with active site a.a residues, and usually Hits the similar type of compounds.

After *in-silico* based screening the top-ranked candidates were also examined using an *in-vitro* bio-assay screening for the evaluation of *β-glucuronidase* inhibitory activity [34]. Twenty compounds were selected, in which compounds **8** and **17** showed significantly higher inhibitory potential, whereas compounds **1**, **5**–**7**, **10**, **12**–**13**, **18**–**19**, were also exhibited good inhibitory effect however compounds **11,** and **15** showed moderate inhibitory activity against *β-glucuronidase* enzyme, as compared to the standard, (D-saccharic acid 1, 4-lactone).

Eleven potent compounds were further subjected to cytotoxicity assay against the mouse fibroblasts (3T3) cell line, to map their preliminary toxicity profile. The standard inhibitor used for 3T3 mouse fibroblasts cell line was cycloheximide. The potent leads of *β-glucuronidase* inhibitors **1**, **5**, and **19** exhibited completely non-cytotoxic behavior. Whereas compounds **6**–**8**, **10**, **12–13** and **17–18** showed a moderate cytotoxicity.

The obtained results revealed that our potent inhibitors contained functional groups NH, sulfur, and thiourea. This observation was further supported from a recently published U.S. Patent [2012 / 0322797] December 20/2012 [35], which also included urea derivatives, sulfur-containing compounds, methoxy-substituted quinoline derivatives, and halo-substituted phenyl-thiophene compounds. Moreover, we observed the presence of the phenyl ring, hetero-atoms (sulfur and oxygen), NH (in case of urea), amide nitrogen, and oxygen atoms, which were all have important chemical structural features of the Pharmacophore, and have interacted with the amino acid residues of binding region, to establish important non-covalent interactions b/w ligand and receptor, including, H-bonding, H-arene, arene-arene, π-π stacking interactions, Van der waal forces of attraction and dipole-dipole interactions, these all contributed towards the best fit ligand-receptor binding pose and ultimately for potent biological activity.

In the way, bioinformatics computational based techniques have proved to be a useful rational approach towards drug designing and discovery process with improved potency and reduce toxic effects.

## Material and methods

### Structure-based individual pharmacophore mapping of PDB 3LPF

**Fig 6:**
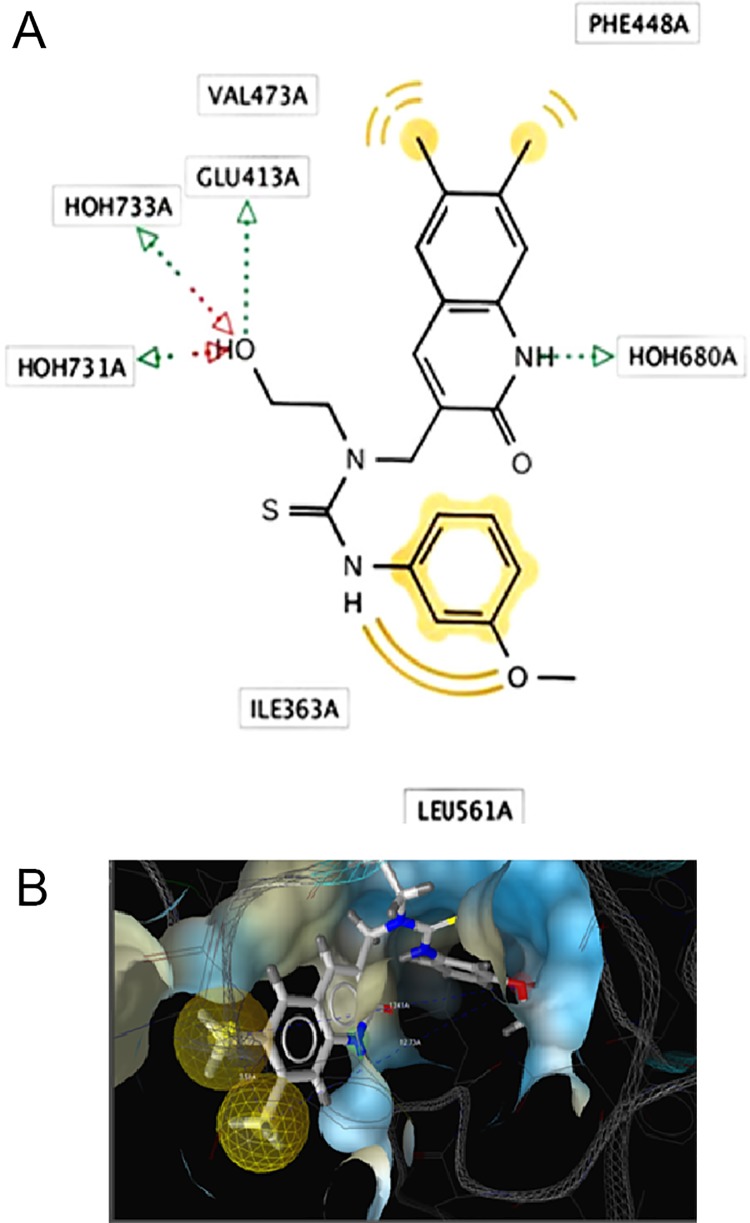
**(A)** The 2D and **(B)** The 3D structure-based Pharmacophore models were derived from PDB I.d 3LPF highlighted with active site surface groove depicted the features. Yellow sphere showed hydrophobic aromatic substituent. Green vector (arrow head) showed the hydrogen bonding of NH with a conserved water molecule HOH 680, another green vector (arrow head) showed the H-bonding of hydroxyl group with amino acid Glu 413A. Two red vectors (arrow heads) showed the H-bonding of HOH 731, and HOH 733 with the hydroxyl group, along with the calculated distances in Å b/w the respective Pharmacophore features.

Two and three-dimensional (2D and 3D, respectively) structure-based Pharmacophore models were derived from PDB I.d 3LPF using Ligand Scout 3.0 version. The software was used to illustrate the following pharmacophore features (Fig 6A and 6B).

Two yellow spheres represented the hydrophobic methyl substituent;

One yellow sphere showed one hydrophobic region due to aromatic substituent;

One green vector (arrow head) depicted the H-bonding of NH with a conserved water molecule HOH 680; another green vector (arrow head) depicted the H-bonding of the hydroxyl group with Glu 413A.

Red vectors (arrow heads) showed the H-bonding of HOH 731 and HOH 733 with the hydroxyl group.

### Structure-based individual pharmacophore mapping of PDB 3LPG

**Fig 7:**
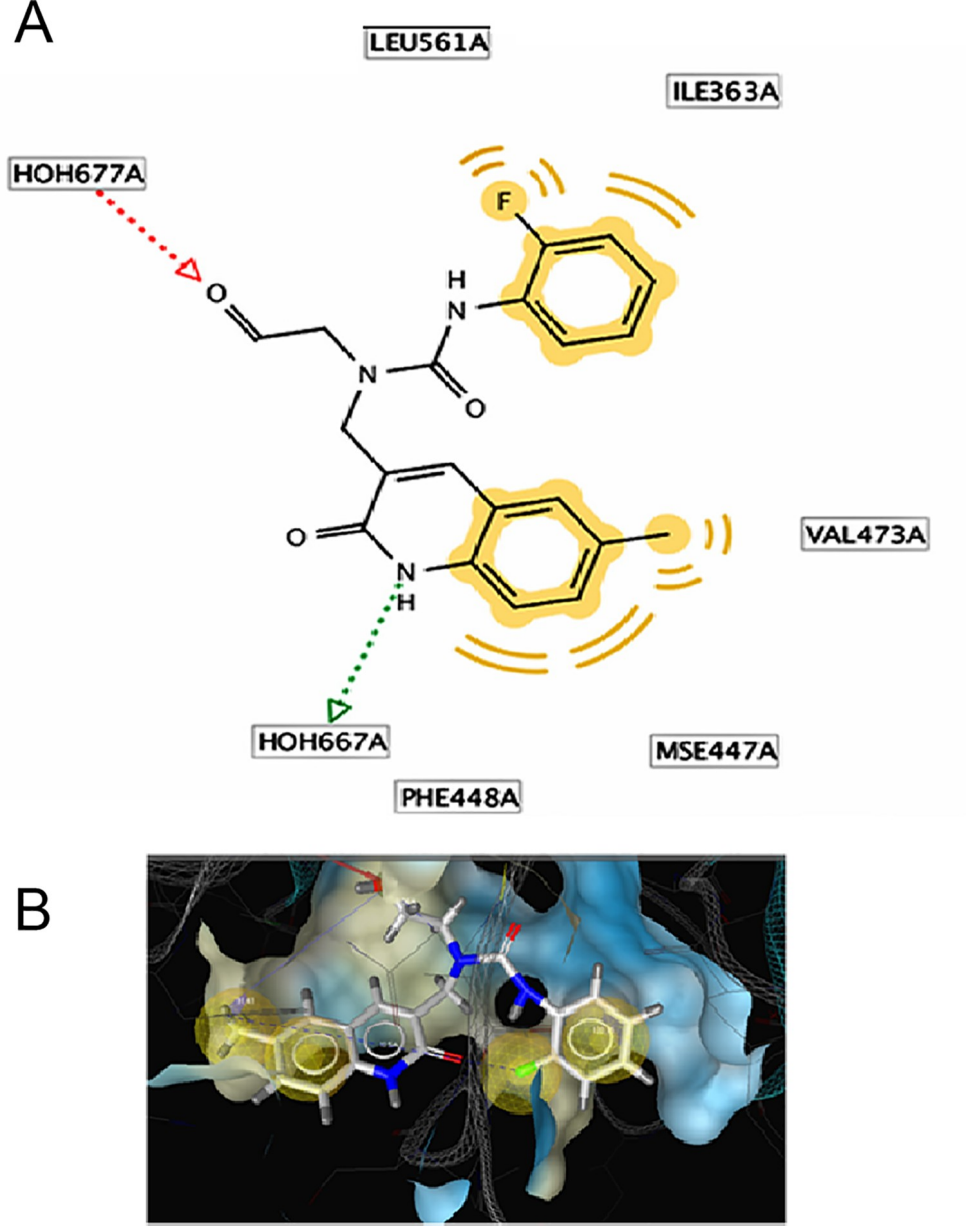
**(A)**The 2D and **(B)**The 3D structure-based pharmacophore models derived from PDB I.d 3LPG highlighted with active site surface groove, depicted the features, two yellow spheres for the hydrophobic methyl benzene ring interacting with the amino acid Val 473A, MSE 447A, PHE 448A. Another two yellow spheres represent the presence of one hydrophobic aromatic ring and hydrophobic fluorine. One green vector (arrow head) showed the H-bonding donor NH to the acceptor HOH 667. One red vector (arrow head) showed the hydrogen donor to the carbonyl oxygen of aldehyde group; along with the calculated distances in Å b/w the respective Pharmacophore features.

The 2D and 3D structure-based pharmacophore models were derived from PDB I.d 3LPG using Ligand Scout 3.0 version. The software depicted the following pharmacophore features (Fig 7A and 7B).

Two yellow spheres represented the hydrophobic region of methyl benzene ring interacting with the amino acids Val 473A, Mse 447A, and Phe 448A;

Another yellow sphere showed hydrophobic aromatic ring and hydrophobic fluorine; the green vector (arrow head) depicted the H-bond donor NH for the acceptor HOH 667;

The red vector (arrow head) represented the hydrogen acceptor of the carbonyl oxygen of aldehyde group from HB-donor HOH677.

### Structure-based individual pharmacophore mapping of PDB 3K4D

**Fig 8:**
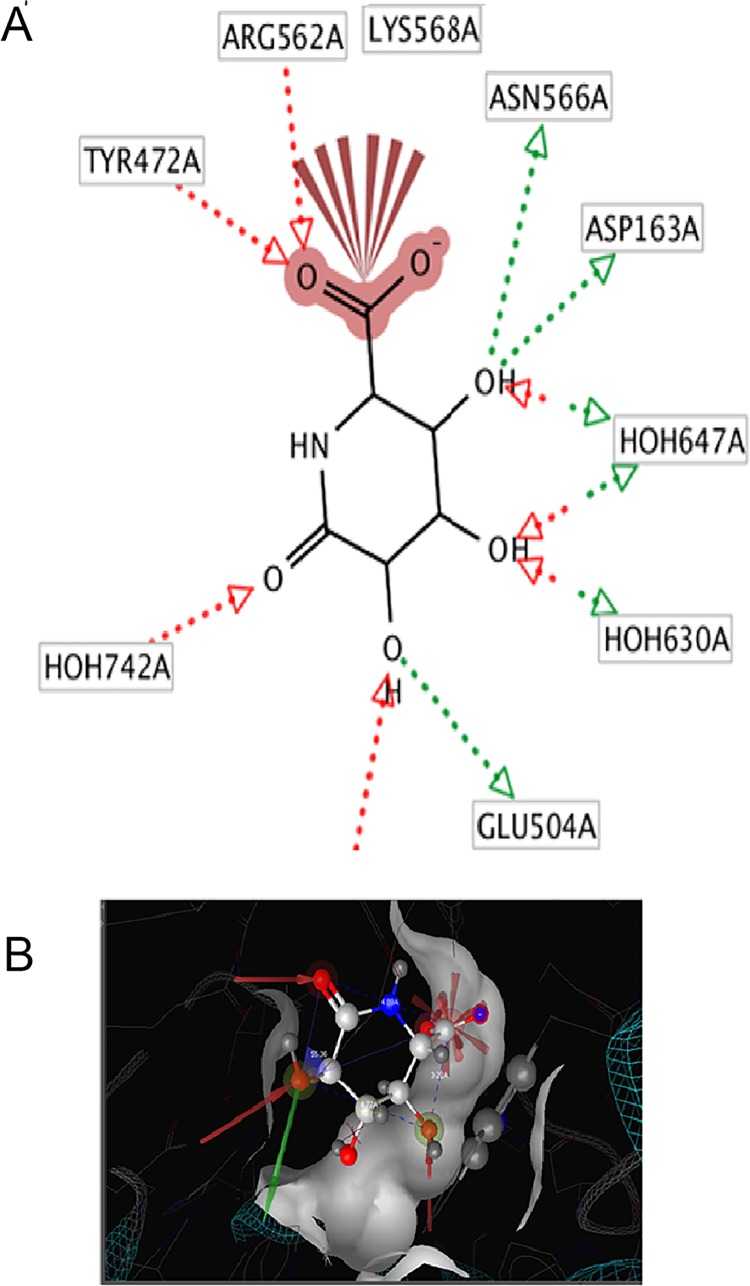
(**A**)The 2Dand (**B**)The 3D structure-based pharmacophore models were derived from PDB I.d 3K4D, highlighted with surface active site groove, depicted the features, two red vectors (arrow heads) for H-acceptors, one for the carboxylate anion, and one for the lactam carbonyl keto group. Three red-green vectors (arrow heads) showed the 3HB-donor / 3HB-acceptor of three OH substituent’s group. One red pointed sphere depicted the negative ionizable area of carboxylate anion; along with the calculated distances in Å b/w the respective pharmacophore features.

The 2D and 3D structure-based pharmacophore models were derived from PDB I.d 3K4D using Ligand Scout software 3.0 versions. The software illustrated the following pharmacophore features (Fig 8A and 8B).

Two red vectors (arrow heads) represented HB-donor from a.a Tyr 472A, Arg 562A to HB-acceptors, one is carboxylate anion (negative ionizable area), while another is lactam carbonyl keto group; three red-green vectors (arrow heads) depicted the 3HB-donor / 3HB-acceptor of three OH groups.

### Generation of structure-based shared and merged feature pharmacophore models

**Fig 9:**
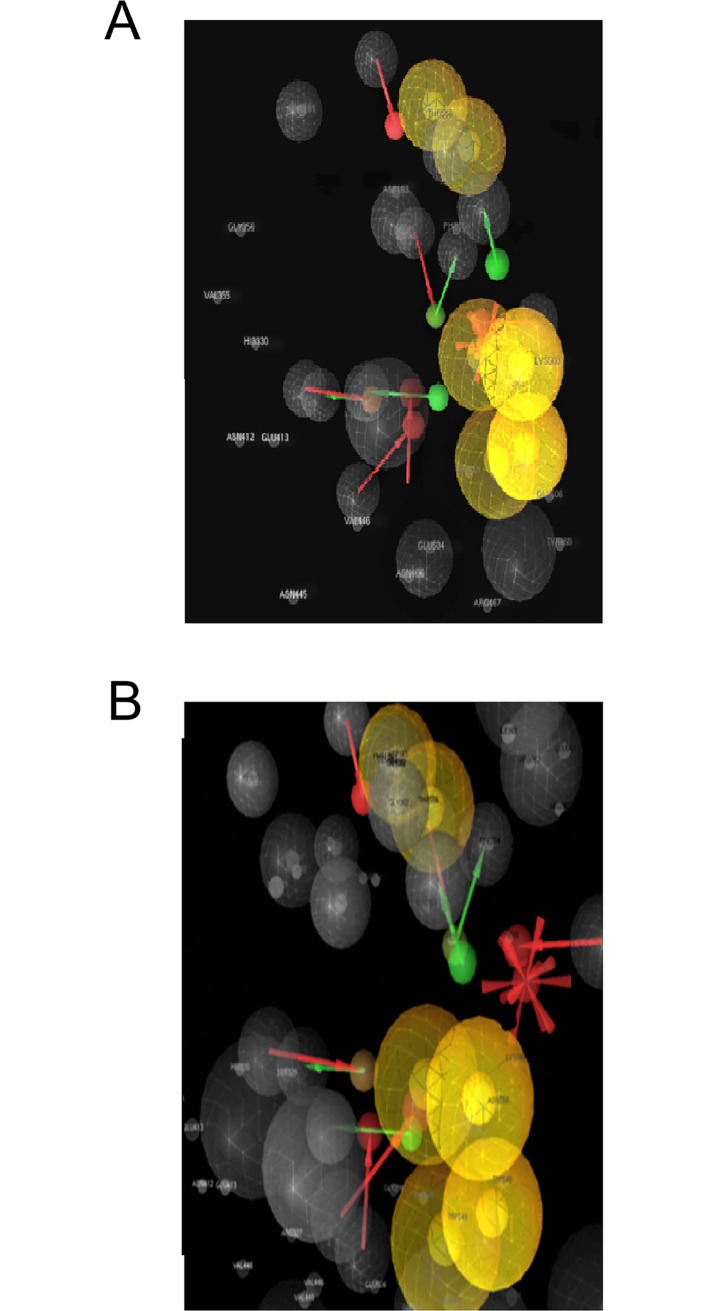
(**A**) It depicts five element based shared feature pharmacophore model derived from PDB I.d, 3LPF, 3LPG and 3K4D by using Ligand Scout, three green vectors showed H-donors, five red vectors showed H-acceptors, four yellow spheres depicted lipophilicity with hydrophobic surface regions grey spheres showed the excluded volume, along with reference point set with a.a residues from active site contour.(**B**): It depicts a merged feature pharmacophore derived model, from PDB I.d 3LPF, 3LPG and 3K4D by using Ligand Scout, it comprised of the features, six red vectors showed the H-acceptors, four green vectors showed the H-donors, one red pointed sphere represented the negative ionizable area, six spheres showed the hydrophobic region, grey spheres depicted excluded volume along with reference point set with amino acid residues within active site contour.

A five-element structure-based shared feature pharmacophore model was also derived. For this purpose, we selected the PDB I.d 3LPF as a reference, to extract the keen binding pattern information’s due to its potent inhibitor bound with IC_50_ value of 326 nM. The remaining two individual derived Pharmacophore models were aligned over it in the alignment pane; finally a shared featured structure-based five element pharmacophore model was generated. Similarly, a merged feature model was also derived by extracting all the Pharmacophore features together. [Fig 9A and 9B].

## Conclusions

In conclusion, our combined methodology of *in-silico* pharmacophore mapping and virtual screening established that computational virtual screening could provide a cost-effective and time saving approach for the selection of *drug-like* candidates. After experimental evaluation of the top ranked identified Hits, we obtained eleven potent inhibitors, three compounds with non-cytotoxic behavior while eight compounds with moderate cytotoxicity against the 3T3 mouse fibroblast cell line. In our designed case study virtual screening Hit results, (scaffold hopping) has been successfully performed, and we identified top 5% enriched *data-base*, contained new classes of compounds with potent biological inhibitory activity against the *β-Glucuronidase* enzyme.

## Supporting information

S1 Data-set It consists of reported inhibitors *data*-s*et*. (DOCX)

S1 Appendix The *in-silico* screening protocol, software’s information, *in-vitro β-glucuronidase* bio-assay screening, and cytotoxicity protocols are included in the supporting appendix, DOI ([https://dx.doi.org/10.17504/protocols.io.q6pdzdn]https://dx.doi.org/10.17504/protocols.io.q6pdzdn). (DOCX)

S2 Appendix Contains supplementary information document of binding curves. (DOCX)
