# Supplementary material for: Bioinformatics: A rational combine approach used for the identification and in-vitro activity evaluation of potent β-Glucuronidase inhibitors
Source: PLoS One. 2018 Dec 5;13(12):e0200502. doi: 10.1371/journal.pone.0200502 (PMC6281186; doi:10.1371/journal.pone.0200502)
Supplement: S1 Data-set — (DOCX) [file pone.0200502.s001.docx]

*β-*Glucuronidase all reported Inhibitors *data-set* used for the molecular docking:

| S.No | Compound Structures | IC_50_, nM/µM |
| --- | --- | --- |
| 1 |  | 17.0 nM |
| 2 |  | 336.0nM |
| 3 |  | 119.0nM |
| 4 |  | 40.7 µM |
| 5 |  | 16.8 µM |
| 6 |  | 15.5 µM |
| 7 |  | 46.1 µM |
| 8 |  | 2.6 µM |
| 9 |  | 1.7 µM |
| 10 |  | 1.9 µM |
| 11 |  | 2.8 µM |
| 12 |  | 3.0 µM |
|  |  |  |
| 13 |  | 4.0 µM |
| 14 |  | 4.8 µM |
| 15 |  | 61.70 µM |
| 16 |  | 6.4 µM |
| 17 |  | 12.10 µM |
| 18 |  | 0.512 µM |
| 19 |  | 0.031 µM |
| 20 |  | 0.046 µM |
| 21 |  | 0.08 µM |
| 22 |  | 0.0016 µM |
| 23 |  | 0.0062 µM |
| 24 |  | 0.0084 µM |
| 25 |  | 59.96 µM |
| 26 |  | 17.3 µM |
| 27 |  | 91.0 µM |
| 28 |  | 16.4 µM |
| 29 |  | 83.0 µM |
| 30 |  | 121 µM |
| 31 |  | 84 .0 µM |
| 32 |  | 6.28 µM |
| 33 |  | 257.1 µM |
| 34 |  | 23.4 µM |
| 35 |  | 276.2 µM |
| 36 |  | 8.9 µM |
| 37 |  | 36.1 µM |
| 38 |  | 94.0 µM |
| 39 |  | 4.23 µM |
| 40 |  | 2.26 µM |
| 41 |  | 19.4 µM |
| 42 |  | 20.1 µM |
| 43 |  | 39.8 µM |
| 44 |  | 22.2 µM |
| 45 |  | 1.1 µM |
| 46 |  | 172.2 µM  198.2 µM |
| 47 |  |  |
| 48 |  | 50.4 µM |
| 49 |  | 2.8 µM |
| 50 |  | 37.7 µM |
| 51 |  | 120.5 µM |
| 52 |  | 5.5 µM |
| 53 |  | 1.17 µM |
| 54 |  | 0.6 µM |
| 55 |  | 1.5 µM |
| 56 |  | 2.1 µM |
| 57 |  | 61.03 µM |
| 58 |  | 44.00 µM |
| 59 |  | 2.10 µM |
| 60 |  | 3.20 µM |
| 61 |  | 30.91 µM |
| 62 |  | 0.72 µM |
| 63 |  | 177.01 µM |
| 64 |  | 10.00 µM |
| 65 |  | 1.88 µM |
| 66 |  | 1.92 µM |
