# Supplementary material for: Bioinformatics: A rational combine approach used for the identification and in-vitro activity evaluation of potent β-Glucuronidase inhibitors
Source: PLoS One. 2018 Dec 5;13(12):e0200502. doi: 10.1371/journal.pone.0200502 (PMC6281186; doi:10.1371/journal.pone.0200502)
Supplement: S1 Appendix — (DOCX) [file pone.0200502.s002.docx]

**S1 Appendix**

**Catalytic a.a residues:**

Catalytic traid amino acid residues include two acidic amino acids [glutamic acid](http://en.wikipedia.org/wiki/Glutamic_acid)  Glu540 and Glu451 anda [tyrosine](http://en.wikipedia.org/wiki/Tyrosine)residue Tyr504. Apart from catalytic traid residues, some other interactive amino acid residues have also been reported which non-covalently interacts with ligand like,Glu413A, Leu361, Phe448A and Tyr472^[^**^5]^.**

**Structure-Based Pharmacophore mapping and Virtual screening:**

According to IUPAC a Pharmacophore can be defined as follows.

“***An ensemble of steric and electronic features that is necessary to ensure the optimal supramolecular interactions with a specific biological target and to trigger (or block) its biological response" it explains how does structurally diverse ligands can bind to a common receptor site. It is an ensemble of steric and electronic features in a molecule that is essential to ensure the optimum interactions with a specific target biological receptor or enzyme protein”* ^[8]^.**

In the present study, five structure-based pharmacophore modelswere derived with the help of three available E.coli *β*-glucuronidase PDBs. 3LPF, 3LPG and 3K4D [Figs 6A-9B] by using Ligand Scout software 3.0 ^[^**^11]^.**

The models were used,to search chemical *data-base* of ICCBS by using Molecular Operating Environment (MOE) (2010-212)^[^**^12^**^].^MOE is the most useable software in computational based drug designing and discovery process designed by Chemical Computing Group. The software is successfully using in structure-based drug design, fragment-based drug design, Pharmacophore mapping, protein and anti-body modeling and 3DQSAR (three dimensional quantitative structure activity relationships). The*Data-base* was at first filtered to obtain *drug-like* candidates by using MOE. Filter removed all of those compounds which deviate from Lipinski rule of five and follow drug ability criteria. After filtration of *data-base*8,262 *drug-like* filtered candidates, were obtained which further used for pharmacophore-based virtual screening. All of the derived models were saved into .Ph4 MOE compatible format. The *data-base* was saved into .mdb MOE compatible format. The software retrieved the Pharmacophore model and searched the query editor to scaffold hopping of chemical database. Pharmacophore-based virtual screening identifiedaltogether 1,249 hits [Fig 2].

**Reported inhibitors *data-set*:**

We selected 66 already reported known inhibitors from the literature of *β*-glucuronidase belongs to different classes of compounds^[13]^ (list provided in supporting information [S1 reported Inhibitors]. All of the compounds 2D .mol format structures were converted into 3D .mol_2_ format by using open babelcommand*-*line.Minimization of compounds was performed by using Omega (OpenEye Scientific Software) to generate low energy conformers^.^

**Molecular Docking:**

Molecular Docking of Pharmacophore based virtually screened 1,249 hits along with 66 reported inhibitors against receptor *β*-glucuronidase was performed by using FRED software, *FRED* uses multi-conformer docking algorithm, which generates a set of low-energy conformers separately, and then do rigid docking of each conformer. *FRED*, is a non-stochastic software and finds the most favorable ligand pose via a systematic exploration of conformational and rotational space Chemgauss-4 is an optimized scoring function of *FRED* Chemgauss-3, The interactions which can be scored by Chemgauss-4 scoring functions includes steric, H-acceptor, H-donors, coordinating groups, metals, lone pairs, polar hydrogens, chelator coordinating groups and overall total H-bonding score between ligand and protein, higher the magnitude of Chemgauss-4 represents the more tightly binding of ligand with receptor through non-covalent forces of interactions,^[^**^15-17]^**.The *data-set* was subjected for docking by using FRED software, which successfully docked 1,315, molecules..

**Enrichment factor:**“*Enrichments smaller than 1 are indicative of a preferred selection of inactive compounds, corresponding to an enrichment of active compounds at the bottom of the sorted library*” ^[^**^19]^**.

E.F was calculated for 5%, 10%, 15% and 20% of docked molecules by using the following formula, [Figs 3A-3D].

Enrichment factor of a (subset) = Hit_s_ / Hit _t_

N_s_ N _t_

Or

**E.F= no of actives in % of *data-base*/% of *data-base** complete *data-base*/total no of known actives**

Where Hit**_s_**= no of actives in a subset, N**_s_** = total no of compounds in a subset, Hit_t_ = total no of actives in the *data-base*, N_t_ = total no of compounds in the data base.

By using the above formula, we calculated the enrichment factor for 5%, 10%, 15% and 20 % for each scoring function of structure-based hits from *in-housedata-base* [Table 1].

**Rescoring by using genetic optimization for ligand docking (*GOLD*):**

As the new version of *FRED*consists ofonly a single consensus scoring function Chemgauss-4.It is the modified form of Chemgauss-3 scoring function.Therefore, to conclude more information rescoring of *FRED* scoring function Chemgauss-4 score was performed by using *GOLD*software 5.1 version ^[^**^20]^**, into Gold-score, Chem-score and ASP (Astex Statistical Potential) scoring functions.Gold scoring functions are dimensionless.However, in each case, the magnitude of score indicates how best the ligand fit/bound with receptor, higher the value of score represents the best binding of ligand with receptor.The Gold score fitness function is the original scoring function of *GOLD*. It deals with ligand binding positions and non-covalent binding interactions such as H-bonding energy, vander waals energy, metal interaction and ligand torsion strain.While, the Chem-score fitness functiondeals with dG that represents total free energy change. It also incorporates a protein-ligand atom clash term and an internal energy term. Chem-score takes account of hydrophobic-hydrophobic contact area, hydrogen bonding, ligand flexibility and metal interaction. After rescoring comparative enrichment factor was calculated for 5%, 10%, 15% and 20% of each scoring function. It was observed that out of these four scoring functions, Chemgauss-4 was found to be as a best one within 5% enrichment of data-base, while for the rest of 10%, 15% and 20% scoring function,Chem-score was found to be the dominant one, comparative to GOLD score and ASP score.(Astex Statistical Potential) [Table 1].

***β-Glucuronidase* inhibition assay protocol:**

*β*-Glucuronidase inhibitory activity was determined with the help of spectrophotometric method by measuring the absorbance at 405 nm of *p*-nitrophenol formed from the substrate (*p*-nitrophenyl-*β*-D-glucuronide, N1627-250 mg, Sigma Aldrich). The total reaction volume was 250 μL. The compound dissolved in DMSO (100%), which becomes 2% in the ultimate assay (250 μL) and the similar conditions were used for standard (D-saccharic acid 1, 4-lactone, Sigma Aldrich). The reaction mixture contained 185 μL of 0.1 M acetate buffer, 5 μL of test compound solution, 10 μL of (1U) enzyme solution (G7396-25KU, Sigma Aldrich) was incubated at 37°C for 30 min. The plates were read on a multiplate reader (SpectraMax plus 384) at 405 nm after the addition of 50 μL of 0.4 mM *p*-nitrophenyl-*β*-D-glucuronide. All assays were performed in triplicate. IC_50_values were calculated by using EZ-Fit software (Perrella Scientific Inc., Amherst, MA, U.S.A.). These values are the mean of three independent readings **^[23]^ [**Table 2].

**Cytotoxicity assay Protocol:**

Cytotoxic activities of eleven potent inhibitors were evaluated in 96-well flat-bottomed micro plates by using the standard MTT (3-[4, 5-dimethylthiazole-2-yl]-2, 5-diphenyl-tetrazolium bromide) colorimetric assay ^[^**^24]^.** For this purpose, 3T3 ( mouse fibroblast)cells were cultured in Dulbecco’s Modified Eagle Medium, supplemented with 5% of fetal bovine serum (FBS), 100 IU/ml of penicillin and 100 µg/ml of streptomycin in 75 cm^2^ flasks, and kept in 5% CO_2_ incubators at 37^o^C. Exponentially growing cells was harvested, counted with haemocytometer and diluted with a particular medium. Cell culture with the concentration of 5x10^4^cells/ml was prepared and introduced (100 µL/well) into 96-well plates. After overnight incubation, medium was removed and 200 µL of fresh medium was added in different concentrations of compounds (1-30µM). After 48 hrs, 200 µL MTT (0.5 mg/ml) was added to each well plate and incubated further for 4 hrs. Subsequently, 100µL of DMSO was added to each well. The extent of MTT reduction to formazan within cells was calculated by measuring the absorbance at 540 nm, using a micro plate reader (Spectra Max plus, Molecular Devices, CA, USA). The cytotoxicity was recorded as concentration causing 50% growth inhibition (IC_50_) for 3T3 cells. The percent inhibition was calculated by using the following formula.

% inhibition = 100-((mean of O.D of test compound – mean of O.D of negative control)/ (mean of O.D of positive control – mean of O.D of negative control)*100).The results (% inhibition) were processed by using Soft- Max Pro software (Molecular Device, USA) **^[24][^**Table 3].
