# Supplementary material for: Bioinformatics: A rational combine approach used for the identification and in-vitro activity evaluation of potent β-Glucuronidase inhibitors
Source: PLoS One. 2018 Dec 5;13(12):e0200502. doi: 10.1371/journal.pone.0200502 (PMC6281186; doi:10.1371/journal.pone.0200502)
Supplement: S2 Appendix — (DOCX) [file pone.0200502.s003.docx]

**S2 Appendix:**

**Supplementary Information**

Percent inhibition binding curves for compounds **1**, **5**-**8**, **10**, **12-13**, and **17**-**19** at different concentrations between 0.78 to 200 μM.
